# Longitudinal influence of COVID-19-related stress on sexual compulsivity symptoms in Chinese undergraduates

**DOI:** 10.1186/s12888-021-03369-x

**Published:** 2021-07-27

**Authors:** Jianjun Deng, Tsingan Li, Jiali Wang, Limei Teng

**Affiliations:** 1 Computer network information center, Chinese academy of sciences, Beijing, 100190 China; 2 Inner Mongolia Honder College of Arts and Sciences, Hohhot, 010070 China; 3 Faculty of Psychology, Beijing Normal University, Beijing, 100875 China

**Keywords:** COVID-19, Depression, Anxiety sexual compulsivity symptom

## Abstract

**Background:**

The coping theory shows that stressful life events are associated with individuals’ psychology/behaviors; meanwhile, the coronavirus disease of 2019 (COVID-19) pandemic is known to have impacted individuals’ physical and mental health. Prior studies revealed that undergraduates have many sexual behavior and emotion disorders, which may be impacted during an isolation period, such as the one brought by COVID-19. However, few studies have explored the longitudinal associations between COVID-19-related stress and sexual compulsivity symptoms (SCS), and the mediating effect of emotions (i.e., depression and anxiety) on this relationship. This longitudinal study aimed to investigate these associations.

**Methods:**

We employed a cross-lagged design (2020/2/12: Time 1, 3219 participants; 2020/6/6: Time 2, 2998 participants) and recruited Chinese undergraduates through an online system to respond to a survey.

**Results:**

Our results showed that COVID-19-related stress at Time 1 directly influenced SCS at Time 1, and there was an indirect influence via depression and anxiety at Time 1. COVID-19-related stress at Time 1 positively correlated with depression, anxiety, and SCS at Time 2, and the first could directly and positively predict SCS at Time 2. Moreover, albeit depression at Time 2 was negatively linked to SCS at Time 2, anxiety at Time 2 enhanced the effect of COVID-19-related stress on SCS.

**Conclusions:**

Our findings extend the literature on SCS, showing that the higher the COVID-19-related stress, the higher the SCS, and the longer-lasting effect was associated with anxiety in undergraduates. Furthermore, depression does not mediate the relationship between COVID-19-related stress and SCS.

## Background

A growing number of studies have found that undergraduates have high levels of sexual compulsivity symptoms (SCS) and have highlighted their impact on individuals’ physical and mental health [1–3]. SCS comprise individuals’ sexuality and sexting behaviors and sexual behavioral intention. Increasingly, young college students have been experiencing a number of psychological and behavioral disorders that are influenced by many factors, such as life events, negative stress, and coping strategies [4–7]. Concurring, the ecological systems theory [8] has pointed out that people’s SCS are influenced by many factors, which can be summarized into two categories: outside environmental factors and individual coping strategies [9–11].

In China, the outbreak of the coronavirus disease of 2019 (COVID-19) has greatly impacted individuals’ physical and psychological health. Particularly, most undergraduates became isolated, be it either in their homes or in diminished school environments, leading to a lack of interrelationships with society; this can be designated as a negative life event. According to the coping theory, negative life events influence individuals’ psychological states and behaviors [12–14]. For example, young people who experience negative life events tend to have a higher SCS [9, 10], and undergraduates may present more impulsive sexual behaviors and psychological states owing to negative life events [1, 2, 4, 15]. However, the underlying mechanism behind the effect of negative life events on people’s sexual behaviors and psychology have yet to be more thoroughly explored.

Based on the stress theory, a person’s coping ability is influenced by stressful life events [16], with negative life or huge stressful events being able to influence SCS [13, 14, 17–19]. Therefore, we hypothesized that COVID-19-related stress would correlate with SCS.

In view of aforementioned findings, the literature provides evidence not only on the direct effect of life events on individual behaviors and psychology, but also on the indirect effect of the first on the latter via coping style [20, 21] and via depression and anxiety [22]. Moreover, studies have shown that emotion can explain the mechanism behind the association between stress and coping [23]; the effect mechanism of emotion on coping models [24]; that emotion regulation strategies are important to influence the effect of stressful life events on individual behaviors [23, 25]; and the effect mechanism of emotions and social culture on individual sexual behavior and motion [26–28]. Additionally, studies have found that depression and anxiety are associated with sexual behavior, and the consumption of sexual content and pornography in undergraduates [29, 30]. However, the correlation and effect mechanisms of stressful life events, emotion, and sexual behavior have yet to be explored.

A longitudinal study showed the importance of experiencing life changing events, as they were strongly associated with individuals’ sexual behaviors [31]; another study noted that longitudinal research is the best method for analyzing people’s sexual developmental patterns [32]. Nonetheless, there is a scarcity of literature on the relationship between COVID-19-related stress and SCS, particularly regarding the potential longitudinal effects.

Along with the COVID-19 outbreak, the Chinese government enforced a range of quarantine policies; particularly, the isolation policy impacted the mode of living and the mental health of most Chinese people, having a unique social significance within the period. Prior studies have suggested that social policies and cultural factors can impact people’s psychology and behavior [15], especially when interrelationships are affected, highlighting that young undergraduates may be eager to communicate with others, peers, or the opposite sex. However, in events that evoke the need for isolation (e.g., the COVID-19 pandemic), they become forced to isolate or remain in a diminished school environment, having to bear with the strains evoked by such stressful events. Amid this, undergraduates may also suffer with the stress that this isolation imposes on their sexuality, including both sexual psychology and behavior.

The cognition-motivation theory shows that life events influence individuals’ emotions and behaviors [33], and based on the coping model, emotion recognition has a relational meaning, which thereby influences individuals’ motivations and desires; these, in turn, evoke emotions, which thereafter influence individuals’ innate behavioral tendencies, and these tendencies tend to be consistent with how people cope. Hence, the literature shows that individuals’ behaviors are consistent with their individual characteristics and with the environments in which they live; namely, SCS may be influenced by both life events and individuals’ emotional factors.

Although previous studies have revealed that COVID-19-related stress influences individual psychology and behavior [34], and assumed that COVID-19-related stress was associated with SCS (as were other negative life events), the emotion recognition theory indicates that emotion can also play a mediating role in the relationship between stressful life events and SCS. Meanwhile, fewer studies have examined the longitudinal influence of emotion on the relationship between COVID-19-related stress and SCS [35].

**Fig. 1:**
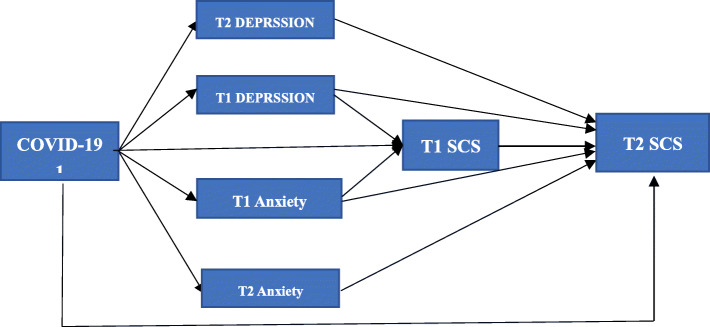
hypothesized mediation model

Therefore, based on the perspective of coping and social psychology, the current study aimed to longitudinally investigate the effect of COVID-19-related stress on SCS. We used a cross-lagged analysis to explore the link between COVID-19-related stress (Time 1) and SCS (Time 2), and the mediating role of depression and anxiety (at Time 1 and 2) in this link (Fig. 1); we hypothesized that depression and anxiety would mediate the link. We believe that our findings are significant for both the literature and society, as follows: for the latter, they can help promote undergraduates’ physical and mental health; for the former, they can serve as references for the development of theories on positive psychology and for the better understanding of negative life events and their long-term effects on individuals.

## Methods

### Participants and procedure

Study participants were undergraduate students from five universities in China who were recruited online via a smartphone app—an epidemic network reporting system of the universities. Each one of the five universities was from a different region in China (east, west, north, south, and middle). Both students living at home and on campus were included in the study. The questionnaires were completed on February 12, 2020 (Time 1) and June 6, 2020 (Time 2). In total, 3219 undergraduates participated at Time 1; after 4 months (Time 2), only 2998 of these undergraduates completed the questionnaires. In Time 2, 730 of the participants were male (24.3%) and 2268 were female (75.7%); their age range was 17–24 years (*M* = 20.5, *SD* = 1.6); 4 people reported belonging to sexual minorities (e.g., gay/homosexual, bisexual, same gender loving, or men who have sex with men, *n* = 4; 0.13%), and 2994 were heterosexual (99.87%). All questionnaires were anonymously completed twice, with participants being identified via specific codes that were individually assigned at Time 1. Since this was a longitudinal study, we only included in the analyses the data of the undergraduates who completed the questionnaire twice; we found no significant differences between the participants who were lost to follow-up and participants included in the analyses, in regard to the COVID-19-related stress and SCS variables.

The design and procedures of this research were concordant with the ethical standards set forth by the research Committee of Beijing Normal University. First, participants read the instructions of the study and its procedures, which included the questionnaire content and informed consent. In the instructions, participants were informed that participation in the study was voluntary, and if they wished to proceed they would have to provide consent.

### Measures

#### COVID-19-related stress

It was assessed using the COVID-19-stressing questionnaire, an 8-item, self-rated questionnaire that examines individual psychological behaviors regarding COVID-19-related stress (e.g., “I take my time to be careful and not get infected with COVID-19,” “I feel more and more nervous as my body temperature rises,” “I fear other people once I know that they were infected with COVID-19,” “I feel bored when everyone is talking about COVID-19”). It is responded through a 5-point Likert scale, ranging from 1 (*lowest*) to 5 (*highest*), with higher scores indicating higher COVID-19-related stress. In this study, the following were the parameters related to validity, reliability, and goodness-of-fit for this scale: Cronbach’s α = 0.772, and *χ*^2^ (6.925)/*df* (4) = 1.731, *p* = 0.000, CFI = 0.998, TLI = 0.988, RMSEA = 0.025. These indicated that this measure had good reliability and validity.

COVID-19-related stress questionnaire was developed using items derived from the Life Event Scale (LES. 1986). In addition, the original questionnaire was preliminary used on a sample comprising 260 undergraduate students, and the results showed that the COVID-19–related stress questionnaire displayed good overall reliability and validity.

#### Depression

It was measured using the Zung Self-rating Depression Scale (SDS), a 20-item scale that assesses individuals’ depression in the last 2 weeks. It is rated on a 4-point scale, ranging from 1 (*no or very little time*) to 4 (*most or* a*ll the time*), and the sum score reflected undergraduates’ depression levels—higher scores denoted higher depression. In this study, the following were the parameters related to validity, reliability, and goodness-of-fit for this scale: Cronbach’s α = 0.959, and *χ*^2^ (158.72849)/*df* (34) = 4.668, *p* = 0.000, CFI = 0.990, TLI = 0.982, RMSEA = 0.057.

#### Anxiety

It was measured using the Zung Self-rating Anxiety Scale (SAS), which is a 20-item scale that examines individuals’ psychological anxiety feelings in the last 2 weeks. It is rated on a 4-point scale, ranging from 1 (*no or very little time*) to 4 (*most or all the time*), and reverse questions were scored in reverse. In this study, the following were the parameters related to validity, reliability, and goodness-of-fit for this scale: Cronbach’s α = 0.886, and *χ*^*2*^ (81.798)/*df* (71) = 1.152, *p* = 0.000, CFI = 0.960, TLI = 0.956, RMSEA = 0.045. These indicated that the scale had good reliability and validity.

#### SCS

We used the Sexual Compulsivity Scale [36] to assess SCS, which is a 10-item measure used to evaluate sexuality-related problems, including people’s sexual thoughts, sexting and sexual behaviors, and sexual behavioral intention (e.g., “My sexual thoughts and behaviors are causing problems in my life,” “My desires to have sex have disrupted my daily life,” and “I sometimes get so horny I could lose control”). It is rated on a 4-point scale (1 = *Not at all like me*; 4 = *Very much like me*), and the sum score reflected SCS levels—higher scores denoted more SCS. In this study, the following were the parameters related to validity, reliability, and goodness-of-fit for this scale: Cronbach’s α = 0.815, and *χ*^2^(82.825)/*df* (17) = 4.872, *p* = 0.000, CFI = 0.990, TLI = 0.982, RMSEA = 0.057.

#### Sociodemographic and COVID-19-related data

Participants were asked to provide some demographic information, including gender, sexual identity (gay/homosexual, bisexual, straight/heterosexual, same gender loving, men who have sex with men, or other), age, and place of residence; and information on their status regarding the COVID-19 pandemic, including whether they lived either with confirmed or suspected COVID-19 patients, and whether they were in home-isolation or hospital control-isolation.

Moreover, since we aimed to assess undergraduates’ SCS, we deemed that participants’ sexual identities would not be of great influence in the variable of interest.

### Statistical analysis

We used SPSS 22.0 and Mplus 7.0 for data analysis. First, we performed descriptive statistics and correlation analyses. Then, while controlling for sex and age, we conducted the structural equation modeling method to investigate the mediation of depression and anxiety in the relationship between COVID-19-related stress and SCS. Finally, we selected bias-corrected (BC) bootstrapping, a non-parametric resampling procedure, to test potential indirect effects; this was conducted based on prior research [37]. When zero was not in the 95% confidence interval (CI), the indirect effect would be significantly different from zero at a *p* < 0.05 (two-tailed).

## Results

### Common method variance

To test the potential effect of common method variance, we included a procedural methodology (i.e., all questionnaires were completed anonymously; all questionnaires had good reliability and validity, serving to reduce or avoid systematic errors as much as possible; some items were scored in reverse in the questionnaires; and the sample was recruited from different universities) and used the Harman’s single factor test. Results showed that there were seven factors with eigenvalues of more than 1, with the first factor having explained 24.84% of the variation, which is less than the critical value of 40% [38]. Thus, there was not a significant common method variance in this study.

### Descriptive statistics and correlation analyses

The descriptive statistics and correlation analyses are reported in Table 1. Participants’ gender correlated only with depression, anxiety, and SCS at Time 2. Meanwhile, participants’ age correlated with COVID-19-related stress only at Time 1. At Time 1, albeit COVID-19-related stress was positively correlated with depression and anxiety, it was negatively correlated with SCS. At Time 2, COVID-19-related stress, depression, and anxiety were all positively related to SCS; however, all variables were negatively related to depression at Time 2. Moreover, COVID-19-related stress, depression, and anxiety at Time 1 were all positively related to SCS at Time 2; however, SCS at Time 1 was negatively related to SCS at Time 2.

**Table 1:**
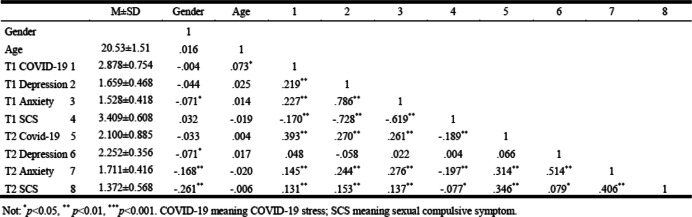
The descriptive and correlation analyses (*n* = 2994). Not: *p < 0.05, ***p = 0.001. COVID-19 meaning COVID-19 stress; SCS meaning sexual compulsive symptom

Namely, COVID-19-related stress, depression, and anxiety affected individuals’ SCS at both periods. Meanwhile, having higher COVID-19-related stress at Time 1, and having higher anxiety at Time 1, denoted that individuals would have higher SCS.

### Mediating effect analyses

**Fig. 2:**
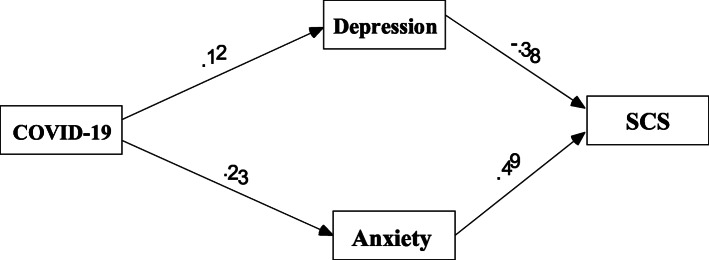
Paths of the mediation model. Note: COVID-19 related stress (COVID-19), Sexual compulsive symptom (SCS). Standardized regression weights are presented. Direct effects and indirect effects are significant. ^*^p < .05, ^**^p < .01, ^***^p < .001

In Model 1, we tested the mediating effect of depression and anxiety in the studied relationship at Time 1. We conducted confirmatory factor analysis to test the goodness-of-fit, with the results being as follows: *χ*^2^ (1.322)/*df* (1) = 1.322, *p* = 0.000, CFI = 1.000, TLI = 1.000, RMSEA = 0.010. Albeit COVID-19-related stress did not directly influence SCS (−.011, *p* = 0.250; CI [− 0.029 0.008]), it did indirectly influence SCS via depression (−.046, *p* = 0.000; CI [− 0.102–0.028]) and anxiety (.113, *p* = 0.000, CI [0.063 0.205]). Thus, at Time 1, COVID-19-related stress negatively influenced SCS via the mediation of depression and anxiety; depression buffered the effect of COVID-19-related stress on SCS; and anxiety facilitated the effect of COVID-19-related stress on SCS (Fig. 2).

In Model 2, which Time 1 COVID-19-related stress and two times’ depression, anxiety effect on SCS (Time 1, Time 2), and depression, anxiety, and Time 1 SCS as mediating roles between Time 1 COVID-19 and Time 2 SCS. By estimating the mediation model with a multi-group analysis, we found no significant difference between Time 1 and Time 2, nor between the male and female groups, for all paths and variances that were examined. Thus, we could test the relationship in the final analyses, while controlling for sex and age. We also conducted a relevant analysis on the other demographic and sociological information collected, and we found that it was not significantly related to stress, depression, anxiety and SCS, and it was not controlled in the SEM analysis.

**Fig. 3:**
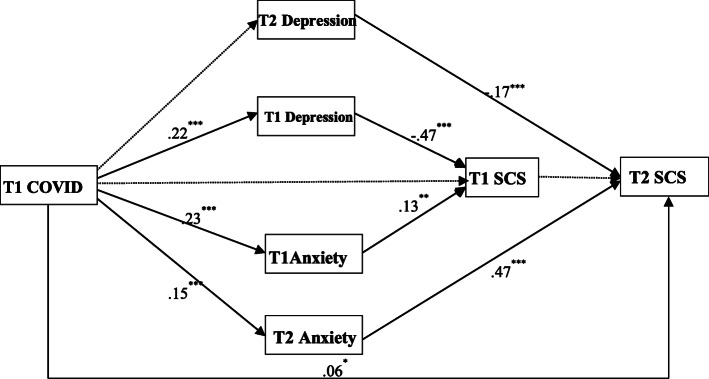
Significantly direct/indirect paths of the mediation model. Note: COVID-19 stress (COVID-19), Sexual compulsive symptom (SCS), TI (Time 1), T2 (Time 2), Standardized regression weights are presented. Direct effects and indirect effects are significant. *p < .05,**p < .01, ***p < .001. The solid line meaning the effect is significant and the dotted line is not significant

We used Mplus 7.0 to test the goodness-of-fit of the hypothetical model. The confirmatory factor analysis results were as follows: *χ*^2^(7.790)/*df* (8) = .974, *p* = 0.000, CFI = 1.000, TLI = 1.000, RMSEA = 0.007; these showed that the hypothetical model was predicted by the data. COVID-19-related stress at Time 1 directly predicted depression at Time 1 (*β* = .22, *p* < 0.001), anxiety at Time 1 (*β* = .23, *p* < 0.001) and Time 2 (*β* = .15, *p* < 0.001), and SCS at Time 2 (*β* = .06, *p* < 0.05). Meanwhile, depression at Time 1 negatively predicted SCS at Time 1 (*β* = −.47, *p* < 0.001); anxiety at Time 2 predicted SCS at Time 1 (*β* = .13, *p* < 0.001); depression at Time 2 negatively predicted SCS at Time 2 (*β* = −.17, *p* < 0.001); and anxiety at Time 2 positively predicted SCS at Time 2 (*β* = .47, *p* < 0.001) (Fig. 3).

Further, we examined the mediating effect by bootstrapping with 5000 samples; since there were no zeroes in the CI, the mediating effects were shown to be significant. Results showed that COVID-19-related stress at Time 1 directly predicted SCS at Time 2 (.055, *p* = 0.030, CI [0.005 0.105]), and indirectly positively predicted SCS at Time 2 via anxiety at Time 2 (.068, *p* = 0.002, CI [0.024 0.111]) (Table 2).

**Table 2:**
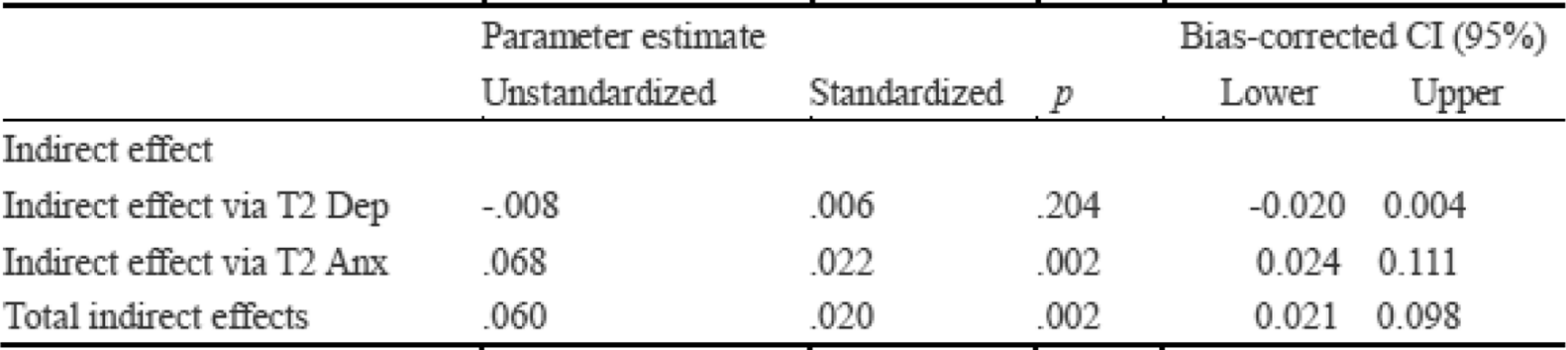
Indirect Effects of COVID-19 on Sexual Compulsive Symptom

Summarizing, our findings revealed that anxiety and depression have different effects in the relationship between COVID-19-related stress and SCS, with only anxiety having a longer-lasting mediating effect on the relationship between the two.

## Discussion

We utilized a cross-lagged design to examine the longitudinal associations between COVID-19-related stress, depression, anxiety, and SCS. Particularly, we examined the mediating role of depression and anxiety in the relationship between COVID-19-related stress and SCS in Chinese undergraduates. Our results revealed the following: that higher COVID-19-related stress was associated with an increase in SCS; that COVID-19-related stress enhances anxiety and SCS; that anxiety mediates the association between COVID-19-related stress and SCS, being a facilitator of the influence of the first on the latter; and that depression did not have a significant mediating effect in the studied relationship. Thus, COVID-19-related stress predicted long-term SCS, anxiety showed an indirect effect on the relationship between COVID-19-related stress and SCS, and depression did not show such indirect effect.

Our results showed that COVID-19-related stress at Time 1 could significantly, directly, and positively predict undergraduates’ SCS at Time 2; namely, individuals with higher stress in February, 2020, showed higher SCS 4 months later. This finds consonance in the literature, which showed that negative life events associated with SCS [2, 13, 17, 18]. Thus, our study participants, who lived with confirmed or suspected COVID-19 patients, had to bear with higher levels of stress, perceived more negative stress, and dealt with negative emotions (i.e., depression and anxiety). Our findings also generally validated the stress theory and the ecological systems theory [16, 39].

Furthermore, our results showed that the environment (i.e., living in isolation) can influence people’s SCS; this finds consonance in the literature [10]. Specifically, our results suggested that individuals with higher stress have more SCS, concurring with numerous past studies [1–4]. Moreover, since COVID-19-related stress was associated with longitudinal SCS in our results, they confirmed the assumptions put forth by the psychosexual development theory; in it, individuals’ sexual psychology and behavioral intentions are influenced by personal traits and the outer environment [40].

Our results also demonstrated that COVID-19-related stress at Time 1 was positively related to depression and anxiety at Time 1, and to anxiety at Time 2; thus, COVID-19-related stress acted as a negative life event, having a significant effect on individuals’ emotions. This finding concurs with a previous study showing that COVID-19 –related stress is a negative life event, and thus evokes negative emotions [34].

Furthermore, our findings highlighted that anxious undergraduates could experience an enhanced effect of COVID-19-related stress on SCS, whereas depression was negatively associated with SCS, even if it did not significantly mediate the studied relationship. This finding concurs with the ecological systems theory [8], Which states that individuals’ SCS can be influenced by outside events and individual emotions [9–11]. Namely, anxiety and depression are different emotions, and albeit the first can mediate the relationship we examined, both emotions were shown to significantly affect SCS.

Furthermore, as described above, our results showed that anxiety at Time 2 mediated and enhanced the effect of COVID-19-related stress at Time 1 on SCS at Time 2, in that anxious individuals with higher COVID-19 related stress would incur in higher SCS later on. Consistently, studies have shown that anxiety can mediate the relationship between life events or stress and emotion or behaviors [9, 41–44], and that depression could not boost individuals’ coping ability toward stressful life event [45]. These results are in line with those of other studies that showed that individuals’ psychological resources may protect mental health, albeit anxiety symptoms could also decrease individuals’ coping efficacy [24] and ability to cope with negative life events [46]. Notwithstanding, our results also showed that both anxiety at Time 1 and depression at both Times 1 and 2 did not significantly mediate the relationship between COVID-19-related stress at Time 1 and SCS at Time 2.

Our results and discussions generally confirm that COVID-19-related stress can have a significant, positive, and direct effect on Chinese undergraduates’ SCS, and that anxiety acts as a partial mediator in this relationship. Furthermore, we revealed that COVID-19-related stress in February, 2020, was the main factor affecting SCS in June, 2020, with anxiety being an enhancing factor in this association.

When coping with outer stress or stressful life events, we are often very likely to incur in anxiety symptoms, whether we like them or not; during a stressful moment such as the COVID-19 outbreak, our results showed that individuals who have higher levels of perceived COVID-19-related stress may have higher anxiety, leading to more SCS.

### Implications

First, we revealed the longitudinal effect of COVID-19-related stress on SCS; COVID-19-related stress was regarded as a powerful coping factor for mental health, and its longitudinal effect was important for individuals, indicating that COVID-19-related stress had a delayed effect on negative life events.

Second, our findings highlighted that the roles of anxiety and depression in the studied relationship differed; only anxiety mediated the relationship between COVID-19-related stress and SCS. This serves as background knowledge about coping with stressful life events and about people’s psychosexual development, which can be used to inform the development of more suitable public policies.

Finally, our study contributes to the scientific understanding and the promotion of health coping models, as we showed the links between negative stress evoked by life events and anxiety.

### Limitations and directions for future research

First, our sample comprised undergraduate students who were recruited online; namely, it did not include other portions of the Chinese population (children, elderly, and other age people), evoking problems related to the external validity of our results. Meanwhile, this manuscript mainly objective explore the mechanism of COVID-19-related stress on SCS for the vast majority of students, so the participants are heterosexual.

Second, although we employed a longitudinal-design, it only covered a four-month period (2020/2/12–2020/6/6). Consequently, the long-term mechanism of COVID-19-related stress on SCS still needs to be further explored in the following days.

Third, the longitudinal effect of COVID-19-related stress could also affect other important individual variables, such as self-concept, resilience, and sexuality attitude; thus, future studies should explore the effect mechanisms in these alternative variables based on the ecological systems theory.

Fourth, even though support from family, spouse, friends, and the wider community might be particularly important for individuals’ mental health status while coping with a negative life event, such as a pandemic, this has not been measured in this study. Therefore, future studies could focus on these factors.

Finally, during the isolation period evoked by the COVID-19 outbreak, individual psychology and physical health may have been influenced by many factors; a study, for example, shows the influence of interpersonal communication and news media on psychological and physical health [47]. Thus, future research should explore the effect mechanism of isolation from a biopsychosocial perspective.

## Conclusions

Our major conclusion was that COVID-19-related stress may positively predict long-term SCS, with anxiety being able to facilitate the influence of the first on the latter. Further, we found that undergraduates’ perceived COVID-19-related stress was significantly associated with higher levels of SCS and anxiety; thus, stakeholders should pay more attention to the prevention of SCS during isolation periods—such as the one evoked by the COVID-19 pandemic—something that can be done by developing and applying psychological interventions that touch upon undergraduates’ emotion.

## Data Availability

The data that support the findings of this study are available on request from the corresponding author, [J.D.].

## Abbreviations

COVID-19: Coronavirus disease of 2019
SCS: Sexual compulsivity symptoms
SDS: Self-rating Depression Scale
SAS: Self-rating Anxiety Scale
